# Editorial: Keeping the Body in Mind: Scientific Effort in Advocating the Best Outcomes for People Living With Severe Mental Illness

**DOI:** 10.3389/fendo.2021.831933

**Published:** 2022-01-03

**Authors:** Katherine Samaras, David Shiers, Roger Chen, Richard I. G. Holt, Jackie Curtis

**Affiliations:** ^1^ Department of Endocrinology, St Vincent’s Hospital, Sydney, NSW, Australia; ^2^ St Vincent’s Clinical School, University of New South Wales Sydney, Kensington, NSW, Australia; ^3^ Adipose Biology, Clinical Obesity and Nutrition Laboratory, Garvan Institute of Medical Research, Darlinghurst, NSW, Australia; ^4^ Psychosis Research Unit, Greater Manchester Mental Health National Health Service (NHS) Trust, Manchester, United Kingdom; ^5^ Division of Psychology and Mental Health, University of Manchester, Manchester, United Kingdom; ^6^ School of Medicine, University of Keele, Newcastle, United Kingdom; ^7^ Human Development and Health Academic Unit, Faculty of Medicine, University of Southampton, Southampton, United Kingdom; ^8^ Southampton National Institute for Health Research Biomedical Research Centre, University Hospital Southampton National Health Service (NHS) Foundation Trust, Southampton, United Kingdom; ^9^ Mindgardens Neuroscience Network, Sydney, NSW, Australia; ^10^ School of Psychiatry, University of New South Wales Sydney, Kensington, NSW, Australia

**Keywords:** obesity, severe mental illness (SMI), diabetes, cardiac disease, psychosis, metabolism, depression, antipsychotic medication

“I welcome this* Frontiers* series on a research area that really matters to families like mine. Collectively these articles challenge services to take a far more holistic body and mind approach. Mine is a 25 year carer perspective for a family member who now experiences several physical conditions alongside a long term mental health disorder, attends several different services and takes a complex mixture of treatments. The seeds of these difficulties were sewn in the early treatment years with little regard for the complex physical health needs our family would face 25 years on. Hence, I particularly welcome the focus of these articles around early psychosis and the impact of antipsychotic treatments on weight, obesity risk, and the prevention of cardiometabolic and other conditions. However, this is not simply about avoiding future physical disorders and dying prematurely. Discovering your son or daughter has a psychosis is bad enough. But reassurances that the weight gain can be tackled once the psychosis settles, soon sound hollow when your child cannot get into their clothes, are ashamed to be seen by their friends, or have discontinued their medication and relapsed. Perhaps the most important contribution of this *Frontiers* series is to encourage families like mine to expect support for a healthy active life from their very first treatment encounter with mental health services.”

*Dr David Shiers*, Topic Editor and Advocate

We are thrilled to present a Research Topic dedicated to optimising physical health in people with severe mental illness (SMI), particularly addressing obesity through best practice in scientific and clinical endeavours. We invited contributions to promote understanding of the “why?” and “how?” that underlie the premature onset of cardiometabolic disease and the 20-plus years short fall in life expectancy, together with the best ways to treat and prevent these devastating consequences of living with SMI. We were rewarded with a series of papers embracing rigor in basic, epidemiological and clinical science which considered the multiple factors contributing to poor health in people with SMI, the impact of antipsychotic medications on energy dysregulation, weight gain and cardiometabolic complications, the clustering of lifestyle risk behaviours and means to address ensuing health issues.

Three excellent reviews examine the cardiometabolic impacts of medications commonly used to treat SMI. The first comprehensively examined the published literature on psychotropic medications and their impact on dysregulation of weight, lipid and glucose metabolism and blood pressure, referring to the extensive basic and clinical science on causative mechanism (Mazereel et al.). The second focuses on the clinical impacts of clozapine, an antipsychotic medication used for treatment-resistant psychosis, with detailed evaluation of the basic and clinical scientific literature (Yuen et al.). A third review examines the fascinating and novel area of the gut microbiome in severe mental illness with antipsychotic medication exposure, examining the impact of these medications on intestinal dysbiosis and the relationship with systemic inflammation (Sfera et al.). Together, these valuable reviews are opportunities to appreciate the multi-system effects of psychotropic medications on physiological dysregulation and the importance of basic scientific research in finding clinical solutions for these metabolic complications. Only by understanding these mechanisms can we start to develop newer antipsychotics that avoid these side effects or new treatments that can offset these adverse consequences.

Obesity prevalence in SMI was collated from 120 studies from 43 nations (Afzal et al.). Whilst most studies were from high-income nations, the review found studies of people with SMI from lower-income nations who previously have often been overlooked. Just over 60% of people with SMI were overweight or obese, with the highest obesity rates in people from North Africa and the Middle East. Studies with matched controls showed three-fold higher obesity rates in people with SMI. This study highlights that, regardless of income status, people with SMI universally suffer higher obesity greater rates compared to their peers. Another study reported high rates of metabolic complications in first episode psychosis (Smith et al., 2020a), highlighting the need for early preventive intervention.

Given the impact of antipsychotic medications on weight gain and its metabolic complications, it might be reasonable to expect that reducing the dose or discontinuing the antipsychotic or switching to a partial agonist might reverse the weight. It is concerning to learn of negligible weight loss impact of these approaches from an elegant systematic review and meta-analysis of forty controlled trials (Speyer et al.). The study highlights three clinical imperatives: first, application of weight gain-prevention strategies from the initial antipsychotic prescription; second, active intervention throughout antipsychotic prescription when weight gain occurs; and, third, intervention to optimise weight when/if deprescription occurs. As clinicians, it is our duty to prevent and treat the metabolic complications of the medications we prescribe. It is also imperative to ensure shared and informed decision making when prescribing high cardiometabolic risk antipsychotic medications, particularly when these medications are used off-label for insomnia, anxiety or behavioural disturbances. Metabolic disturbances accrued on treatment, even if obesity cannot be mitigated, require intervention to treat and normalise lipids, glucose and blood pressure.

Basic science contributions implicate novel targets within the progesterone receptor in preclinical models for amelioration of antipsychotic-induced metabolic complications (Cao et al.).

Heartening is a growing evidence base for weight gain prevention through early intervention (Smith et al., 2020b), building on that from our “Keeping the Body in Mind Program^”^ (1), from which this Research Topic borrows its name. The 12-week intervention included health behaviour education and facilitated exercise. In a group with high health risk behaviours, there were reductions in smoking and substance abuse and improvements in reported diet and physical activity, without any anthropometric change. Without controls, it was not possible to determine whether weight gain was mitigated by the program, though this might be expected based on naturalistic studies. The study from Smith et al., 2020b indicates the importance of formulating bespoke interventions that meet the specific needs of people with SMI and the importance of empowerment through education, early interventions and advocacy. Frontiers looks forward to further studies of such early interventions aimed at preventing obesity and its associated complications in people with severe mental illness and other groups with special needs.

## Author Contributions

KS: Conception of RT and wrote part of the editorial. DS: wrote part of the editorial and contributed to editing. RC, RH, and JC contributed to editing and revisions of the editorial. All authors contributed to the article and approved the submitted version.

## Author Disclaimer

Views expressed are personal and not those of NICE.

## Conflict of Interest

DS is an expert advisor to the National Institutes of Clinical Excellence (NICE, UK) Centre for Guidelines.

The remaining authors declare that the research was conducted in the absence of any commercial or financial relationships that could be construed as a potential conflict of interest.

## Publisher’s Note

All claims expressed in this article are solely those of the authors and do not necessarily represent those of their affiliated organizations, or those of the publisher, the editors and the reviewers. Any product that may be evaluated in this article, or claim that may be made by its manufacturer, is not guaranteed or endorsed by the publisher.
